# Snake Cathelicidin from *Bungarus fasciatus* Is a Potent Peptide Antibiotics

**DOI:** 10.1371/journal.pone.0003217

**Published:** 2008-09-16

**Authors:** Yipeng Wang, Jing Hong, Xiuhong Liu, Hailong Yang, Rui Liu, Jing Wu, Aili Wang, Donghai Lin, Ren Lai

**Affiliations:** 1 Biotoxin Units of Key Laboratory of Animal Models and Human Disease Mechanisms, Kunming Institute of Zoology, Chinese Academy of Sciences, Kunming, Yunnan, China; 2 Shanghai Institute of Materia Medica, Chinese Academy of Sciences, Shanghai, China; 3 Key Laboratory of Microbiological Engineering of Agricultural Environment, Ministry of Agriculture, Life Sciences College of Nanjing Agricultural University, Nanjing, Jiangsu, China; 4 Graduate School of the Chinese Academy of Sciences, Beijing, China; Massachusetts General Hospital and Harvard Medical School, United States of America

## Abstract

**Background:**

Cathelicidins are a family of antimicrobial peptides acting as multifunctional effector molecules of innate immunity, which are firstly found in mammalians. Recently, several cathelicidins have also been found from chickens and fishes. No cathelicidins from other non-mammalian vertebrates have been reported.

**Principal Findings:**

In this work, a cathelicidin-like antimicrobial peptide named cathelicidin-BF has been purified from the snake venoms of *Bungarus fasciatus* and its cDNA sequence was cloned from the cDNA library, which confirm the presence of cathelicidin in reptiles. As other cathelicidins, the precursor of cathelicidin-BF has cathelin-like domain at the N terminus and carry the mature cathelicidin-BF at the C terminus, but it has an atypical acidic fragment insertion between the cathelin-like domain and the C-terminus. The acidic fragment is similar to acidic domains of amphibian antimicrobial precursors. Phylogenetic analysis revealed that the snake cathelicidin had the nearest evolution relationship with platypus cathelicidin. The secondary structure of cathelicidin-BF investigated by CD and NMR spectroscopy in the presence of the helicogenic solvent TFE is an amphipathic α-helical conformation as many other cathelicidins. The antimicrobial activities of cathelicidin BF against forty strains of microorganisms were tested. Cathelicidin-BF efficiently killed bacteria and some fungal species including clinically isolated drug-resistance microorganisms. It was especially active against Gram-negative bacteria. Furthermore, it could exert antimicrobial activity against some saprophytic fungus. No hemolytic and cytotoxic activity was observed at the dose of up to 400 µg/ml. Cathelicidin-BF could exist stably in the mice plasma for at least 2.5 hours.

**Conclusion:**

Discovery of snake cathelicidin with atypical structural and functional characterization offers new insights on the evolution of cathelicidins. Potent, broad spectrum, salt-independent antimicrobial activities make cathelicidin-BF an excellent candidate for clinical or agricultural antibiotics.

## Introduction

Innate immunity uses gene-encoded antimicrobial peptides to form a first line of host defense against noxious microorganisms [1], [2]. A large amount of antimicrobial peptides have been identified from animals, plants and microorganisms. Several families of antimicrobial peptides including cathelicidin, liver-expressed antimicrobial peptide (LEAP) or hepcidin, histatin, and defensin have been identified from mammalians [3]–[7]. Defensins and hepcidins are characterized by the presence of multiple disulfide bridges, whereas histatins and most of cathelicidins are linear molecules without disulfide bridges.

After the first discovery of cathelicidin (Bac5) from bovine neutrophils, a large amount of cathelicidins have been identified from other mammalians [8]–[13]. As other antimicrobial peptide families, structurally divergent cathelicidins have been found, even in a single mammalian species. For example, there are at least seven cathelicidins in cattle, horse, pig, sheep, and goat [8]. Some exceptions are in human, rhesus monkey, mouse, rat, and guinea pig, only a single cathelicidin was found [8], [14]–[18].

Cathelicidin antimicrobial peptides are released from their corresponding inactive precursors by proteolytic cleavage [8]. The cathilicidin family of proteins is characterized by the presence of a highly conserved anionic cathelin domain [3], [8], [19]. Cathelin is an inhibitor of the cysteine proteinase cathepsin L [20]. In the precursors of cathelicidins, the highly conserved cathelin domains composed of about 100 amino acid residues is flanked by a signal peptide fragment (approximately 30 residues long) on its N-terminus, and by a structurally divergent cationic antimicrobial peptide region on its C-terminus [8]. Upon activation, most of cathelicidin precursors proteolytically cleaved to release the cathelin domain and the C-terminal mature antimicrobial peptides. Some intact cathelicidin precursors are also found in the biological fluids where cathelicidin expressed [3], [21]. Elastase seems to be the most common peptidase to release mature cathelicidins [22], [23]. In human hCAP18, however, protease-3 cleaves the proprotein [24]. Mature cathelicidins can be further degraded by some serine proteases because multiple cationic amino acid residues (Arg or Lys) are in the sequences of cathelicidins [25]. In addition, hCAP18 could be degraded by aspartyl protease (gastricsin) at vaginal pH. Some hydrolytic fragments of cathelicidin were found to possess increased antimicrobial abilities [26].

Recently, several cathelicidins have been identified from some non-mammalian vertebrates including hagfish [27], rainbow trout [28], [29], atlantic salmon [29], and chicken [30], [31]. As the oldest jawless craniates, hagfish lacks adaptive immunity [8], [32]. The presence of cathelicidins in hagfish may indicate that cathelicidin genes appeared early in phylogenesis [8]. Cathelicidins have been found from most of vertebrates including fish, bird, mammalian, whereas no cathelicidins have been found from amphibians and reptiles. In this wok, a cathelicidin from snake was identified and characterized.

## Materials and Methods

### Materials

*B. fasciatus* crude venom and venomous glands were collected from Guang Xi Province, China. The SMART™ PCR cDNA synthesis kit was purchased from Clontech, USA. Chromatography media Sephadex G-50 and CM-Sephadex C-25 were obtained from Amersham Bioscience, Sweden. Trifluoroacetic acid (TFA, HPLC grade) was from Perkin-Elmer. Acetonitrile (ACN, HPLC grade) was bought from Fisher Chemicals. 2,2,2-trifluroethanol-d3 98% (TFE-d3), sodium dodecyl-d_25_ sulfate (SDS-d25) 98.7%, trimethylsilyl-2,2,3,3-tetradeuteropropionic acid (TSP)-d_4_ 98% and D_2_O 99% were purchased from Cambridge Isotope Laboratories. Reverse Phase High Performance Liquid Chromatography (RP-HPLC) C4 column (30 cm×0.46 cm) was from Agilent. The pMD18-T vector was from Takara, Dalian, China. All other reagents were of analytical or sequencing grade. The animals used for the experiments were treated according to the protocols evaluated and approved by the ethical committee of Kunming Institute of Zoology.

### Isolation of cathelicidin-BF

The purification procedure was according to our previously report [33], [34]. 0.4 g *B. fasciatus* crude venom was first fractionated using gel filtration chromatography Sephadex G-50 column (26 cm×100 cm), equilibrated with 50 mM Tris–HCl, 50 mM NaCl (pH 7.8). The elution was performed with the same buffer and monitored at UV absorption of 280 nm. The peak having antimicrobial activity was collected and further dialyzed against PBS (pH 6.0). The dialyzed product was next subjected to the cation-exchange CM-Sephadex C-25 column (1.6 cm×30 cm). The elution was achieved with a linear NaCl gradient, at a flow rate of 1 ml/min. The peak with antimicrobial activity was collected and finally purified by reverse phase high performance liquid chromatography (C4), equilibrated with 0.1% (v/v) TFA/water. The elution was performed with a liner gradient of acetonitrile at a flow rate of 0.7 ml/min.

### Primary structural analysis

The amino acid sequence of the N-terminus was determined by the automated Edman degradation using an Applied Biosystems pulsed liquid-phase sequencer, model 491. Electrospray ionization mass spectrometry (ESI-MS) was used to determine the molecular weight by a Finnigan LCQ ion trap mass spectrometer (ThermoFinnigan, San Jose, CA, USA) in positive-ion mode. The sample solutions (50%H2O/50%ACN) were infused into the mass spectrometer via a Harvard syringe pump (Holliston, MA, USA). The spray voltage was set to +4.5 kV. Spectra were acquired by summing 30 scans.

### CD and NMR spectroscopy

Circular dichroism (CD) spectra were recorded at 298 K on a JASCO J-810 spectrometer (Jasco, Japan). Samples were prepared by dissolving the peptide powder to a concentration of 90 µM in TFE/H_2_O mixtures or in SDS micelles of different concentrations. The spectra were measured between 190 and 250 nm using 0.1 cm path-length cell with 1 nm bandwidth, 1 sec response time, and a scan speed of 100 nm/min. Three consecutive scans per sample were performed, added and averaged followed by subtraction of the signal of the solvent. The secondary structure elements of the peptides were estimated according to the Yang formula [35].

Samples for nuclear magnetic resonance (NMR) measurements contained 4 mM cantheicidin-BF in TFE-d3/H_2_O (9∶1, v/v) at pH 6.5, or in 300 mM SDS-d25 at pH 6.5. All NMR spectra were recorded at 298 K on a Varian Unity INOVA 600 MHz spectrometer equipped with three RF channels and a triple resonance z-axis pulsed-field gradient probe. The 2D ^1^H-^1^H TOCSY spectra were acquired with a mixing time of 75 ms, while ^1^H-^1^H NOESY spectra were acquired with mixing times of 200 and 300 ms. The watergate approach was employed for water suppression. Data were collected with 256 and 1024 complex data points in t1 and t2 dimensions, respectively. Signals were averaged over 64 transients. All NMR spectra were processed and analyzed using the NMRPipe/NMRDraw software and the Sparky program [36], [37]. Linear prediction in the t1 dimension was used before the Fourier transformation. Assignments of the proton resonances were achieved using both TOCSY and NOESY spectra. The ^1^H chemical shifts were referenced to TSP. The secondary structure was predicted using the H_α_ Chemical Shift Index approach [38].

### SMART cDNA synthesis

Total RNA was extracted using TRIzol (Life Technologies, Ltd.) from the venomous glands of *B. fasciatus*. cDNA was synthesized by SMART™ techniques by using a SMART™ PCR cDNA synthesis kit (Clontech, Palo Alto, CA). The first strand was synthesized by using cDNA 3′ SMART CDS Primer II A, 5′-AAGCAGTGGTATCAACGCAGAGTACT (30) N-1N-3′ (N = A, C, G or T; N-1 = A, G or C), and SMART II An oligonucleotide, 5′-AAGCAGTGGTATCAACGCAGAGTACGCGGG-3′. The second strand was amplified using Advantage polymerase by 5′ PCR primer II A, 5′-AAGCAGTGGTATCAACGCAGAGT- 3′.

### Screening of cDNA encoding cathelicidin-BF

The cDNA synthesized by SMART™ techniques was used as template for PCR to screen the cDNAs encoding serine protease inhibitor. Two oligonucleotide primers, BFS_1_ 5′-AA(A/G)TT(T/C)TT(T/C)AG(A/G)AA(A/G)(C/T)T(A/T/C/G)AA(A/G)AA (A/G)-3′, in the reverse direction, a specific primer designed according to the amino acid sequence determined by Edman degradation and primer II A as mentioned in “*SMART cDNA synthesis*” in the sense direction were used in PCR reactions. The DNA polymerase was Advantage polymerase from Clontech (Palo Alto, CA) The PCR conditions were: 2 min at 94°C, followed by 30 cycles of 10 sec at 92°C, 30 sec at 50°C, 40 sec at 72°C. Finally, the PCR products were cloned into pGEM®-T Easy vector (Promega, Madison, WI). DNA sequencing was performed on an Applied Biosystems DNA sequencer, model ABI PRISM 377.

### Expression profile of tissues

Reverse transcription-polymerase chain reaction (RT-PCR) was carried out to analyze gene expression of cathelicidin-BF in *B. fasciatus*. Total RNA extraction from different tissues and first-strand cDNA synthesis were the same as described above. The primers were, forward primer, 5′-cathelicidin: 5′-ATGGAAGGGTTCTTCTGGA AGACC-3′, and reverse primer, 3′-cathelicidin: 5′-CAAATTAGAAGGGGATGGAG ACC-3′. PCR conditions were: 95°C (3 min), and 30 cycles of 95°C (30 s), 56°C (30 s), 72°C (3 min) followed by a 15 min extension period at 72°C. The control PCR was performed using the specific primers (forward primer, actin-s 5′-GGGTGTGATGGT TGGCATGG-3′, and reverse primer, actin-as 5′-TGGCTGGAAGAGGGCTTCTG-3′) for snake actin, using the same conditions as above.

### Alignment and phyogenetic comparison of cathelicidins

Cathelicidin sequences were obtained from the protein database at the National Center for Biotechnology Information. The phylogenetic tree is constructed by neighbor-joining analysis, using the ClustalW program (version 1.8).

### Antimicrobial testing

Antimicrobial activities of cathelicidin-BF and cathelicidin-BF15 (VKRFKKFFRKLKKSV) were tested according to our previous methods [39]–[42]. Ampicillin, benzylpenicillin (Amresco) and Imipenem and Cilastatin Sodium for Injection (ICS, Merck) were used as positive controls. The details were provided in the Materials and Methods S1.

### Bacteria killing kinetics

*In vitro* bacteria killing kinetics of cathelicidin-BF, ICS (its minimal inhibitory concentration (MIC) for *Escherichia coli* 08A866 is 0.15 µg/ml), and HDW (an antimicrobial peptide from the frog of *Rana nigrovittata*, with a amino acid sequence of FIGPVLKIATSILPTAICKIFKKC, its MIC for *E. coli* 08A866 is 18.7 µg/ml), respectively, were determined according to the methods described by Mygind *et al* [43]. The details were provided in the Materials and Methods S1.

### Hemolysis, cytotoxicity, serum stability

Hemolytic activity was checked by incubating the tested samples with human red blood cells to determine hemoglobin releasing ability by measuring the absorbance at 540 nm, using 1% Triton X-100 as a positive control. Cytotoxicity and serum stability were measured according the methods described by Mygind *et al* [43]. The details were provided in the Materials and Methods S1.

### Synthetic Peptides

All of the peptides used for the bioactivity assays and NMR analysis in this paper were synthesized by the peptide synthesizer (433A, Applied Biosystems) in AC SCIENTIFIC (Xi An) INC. (Xi An, China) and analyzed by HPLC and MALDI-TOF mass spectrometry to confirm that the purity was higher than 95%. All peptides were dissolved in water.

## Results

### Isolation of cathelicidin-BF from the snake venoms of *B. fasciatus*

The crude snake venom was separated into four fractions by Sephadex G-50 gel filtration as our previous report (Figure S1a) (Fig. S1a) [33], [34]. The fraction III, containing antimicrobial activity was further subject to CM-Sephadex C-25 cation-exchange column, and nine sub-fractions were collected (Figure S1b). The fraction VI with both trypsin-inhibitory and antimicrobial activities was further purified using RP-HPLC. The peak with antimicrobial activity is marked with an arrow in Figure S1c. The purified antimicrobial peptide was named cathelicidin-BF. The molecular mass and purity of purified cathelicidin-BF was further analyzed by a ESI mass spectrometry, giving a [M+7H]^7+^, [M+7H]^6+^, [M+7H]^5+^and [M+7H]^4+^of 521.1, 607.6, 729.1 and 991.5 (Figure S2), indicating that purified cathelicidin-BF has a molecular weight of 3637.5–3638.5.

### Structure characterization of cathelicidin-BF

Purified cathelicidin-BF was subjected to amino acid sequence analysis using automated Edman degradation. Its amino acid sequence is KFFRKLKKSVKKRAKEFFKKPRVIGVSIPF. Cathelicidin-BF is composed of 30 amino acid residues including 12 basic residues (9 Lys and 2 Arg), 5 phenylalanines, and only one acidic amino acid residue (Glu). It is a lysine-rich and phenylalanine-rich peptide. Analysis using the ExPASy MW/pI tool (http://www.expasy.ch/tools/pi_tool.html) showed that cathelicidin-BF had the predicted pI (isoelectric point) of 11.79 and a predicted molecular weight of 3637.5 that matched well with the observed mass by ESI mass spectrometry (Figure S2). By BLAST search, no similar sequence was found in GenBank.

**Figure 1:**
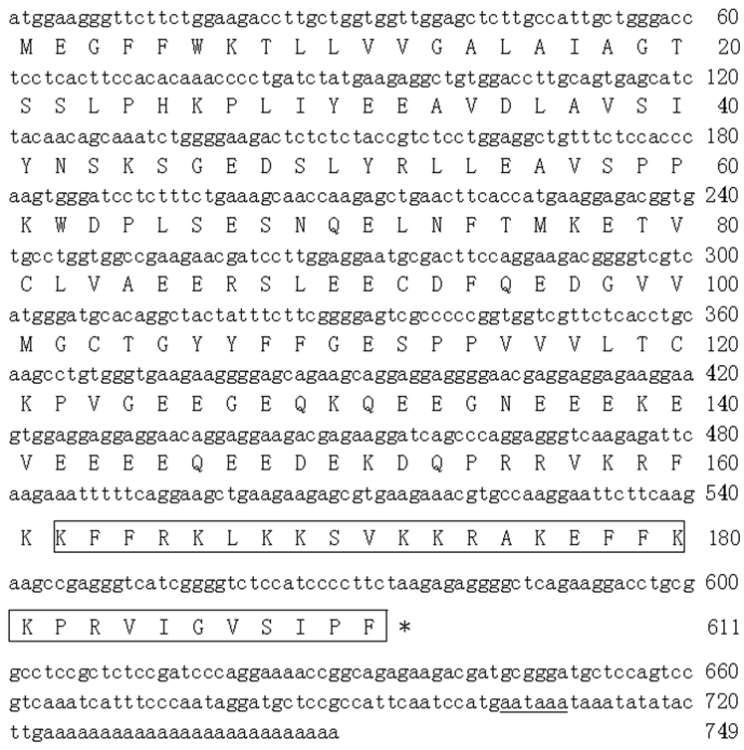
The cDNA sequence encoding cathelicidin-BF and the predicted precursor amino acid sequence. The amino sequence of purified cathelicidin-BF is *boxed*. The stop codon is indicated by a *star* (*). The potential polyadentlation signal (AATAAA) is *underlined*.

Several positive clones, which contained an insert of 750 bp were identified and isolated from *B. fasciatus* venomous gland cDNA library. The complete nucleotide sequence of cDNA (GenBank accession EU753183) and deduced amino acid sequence of cathelicidin-BF precursor are shown in Figure 1. Unexpectedly, the cathelicidin-BF precursor displays the maximal similarity (47%) with predicted myeloid cathelicidin 3 from *Ornithorhynchus anatinus* (GenBank accession XP_001512130) by BLAST search. The protein precursor is composed of 191 amino acid (aa) residues, including a predicted signal peptide, a conserved cathelin domain and a mature cathelicidin-BF. Noticeably, four cysteines that are conserved in the cathelin domain of all mammalian cathelicidins are also invariantly spaced in cathelicidin-BF precursor, suggesting that the snake cathelicidin-BF precursor is a real mammalian cathelicidin.

**Figure 2:**
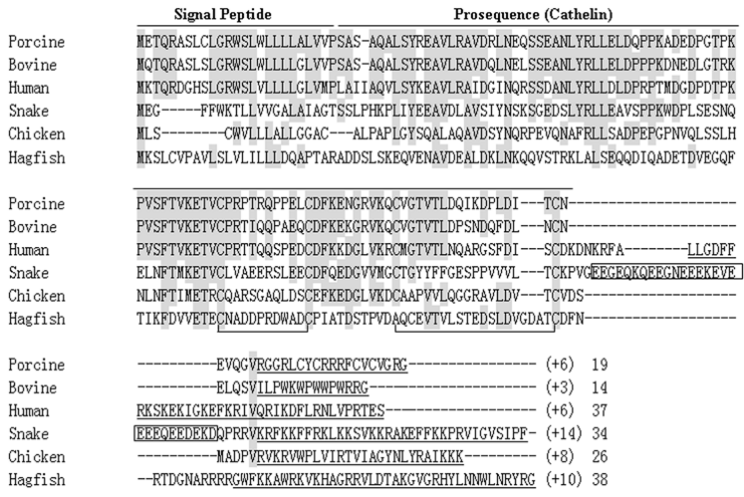
Multiple sequence alignment of snake cathelicidin with other representative cathelicidins. Cathelicidin-BF precursor is aligned with porcine, bovine, human, chicken and hagfish cathelicdins. *Dashes* are inserted to optimize the alignment, and conserved residues are *shaded*. Two intramolecular disulfide bonds in the cathelin pro-sequence are shown. Mature cathelicinds are *underlined*, and their net charge (*in parenthesis*) and length are also indicated. The acidic fragment insertion in cathelicidin-BF is *boxed*.

The amino acid sequence of cathelicidin-BF determined by Edman degradation is identical with the amino acid sequence deduced from the cDNA sequence. There is a possible cleavage site (Valine^157^) for elastase at the N-terminus of the mature cathelicidin-BF (Figure 1). Based on the possible cleavage site, a 34-aa peptide should be released from the precursor, but the purified cathelicidin-BF is only composed of 30 aa. Different from other cathelicidins, there is an acidic doman between the cathelin doman and the antimicrobial peptide in the cathelicidin-BF precursor (Figure 2).

**Figure 3:**
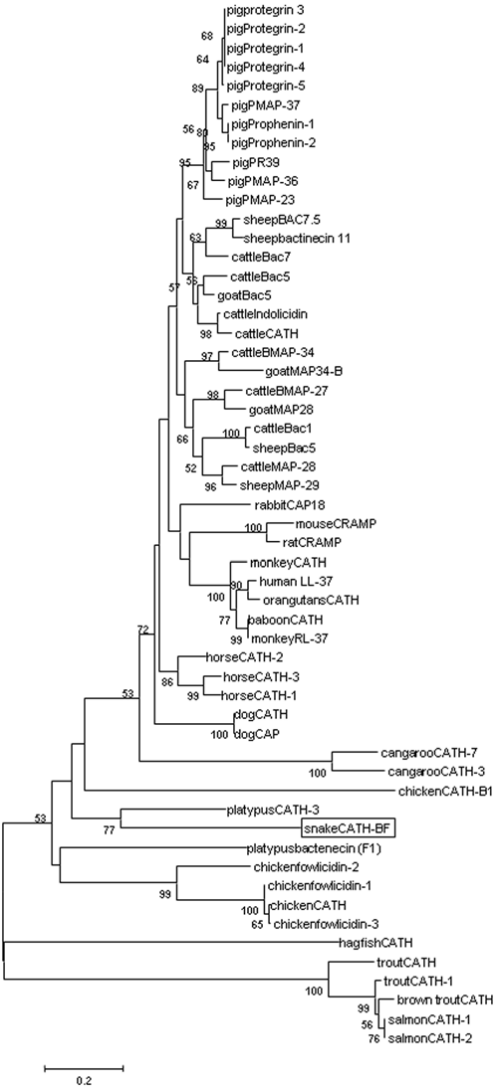
Phylogenetic analysis of cathelicidins. Phylogenetic dendrogram obtained by neighbour-joining analysis based on the proportion difference (p-distance) of aligned amino acid sites of the full-length peptide sequences. Only bootstrap values >50% (expressed as percentages of 1000 resamplings) are shown at branching points. Snake cathelicidin-BF is boxed.

Evolution analysis revealed that all vertebrate cathelicidins formed three distinct clusters with fish cathelicidins located in a separated clade from others. Supported by a bootstrap value of 77%, cathelicidin-BF was clustered with platypus CATH-3 (Figure 3). Although platypus is a mammal, it also has reptilian features, For instance, it lays eggs and it is venomous [44]. The close evolution relationship of cathelicidin-BF found in the venoms of *B. fasciatu*s with platypus cathelicidins may provide further proof for platypus's reptilian features.

### Secondary structures detected by CD and NMR

The secondary structure elements in different solvent environments were detected by CD spectroscopy (Figure S3, Table S1). In H_2_O, the CD spectrum of cathelicidin-BF showed a strong negative band at 200 nm, indicative of a random-coil conformation. Interestingly, in TFE/H_2_O mixtures, the CD spectra showed double minima at 208 and 222 nm, indicating a highly α-helical conformation. The signals at 208 and 222 nm were intensified gradually by increasing concentrations of TFE, which indicated that the helicity of the peptide was increased in more hydrophobic or membrane-mimetic environments. The CD spectra of the peptide in SDS micelles also showed a typical α-helix pattern and the content of the α-helix structure increased with the increasing SDS concentration.

NMR spectra recorded on the peptide in SDS micelles were of low quality and can not be used for structural analysis, might due to aggregation of the peptide in negative charged SDS micelles. Therefore, the helical structure of the peptide in TFE/H_2_O mixture was investigated using NMR spectroscopy. Table S2 lists the nearly complete assignments of the proton chemical shifts of cantheicidin-BF in TFE/H_2_O mixture (9∶1, v/v). Comparison of the HN-HN region of NOESY spectrum recorded on cantheicidin-BF in H_2_O with that in TFE/H_2_O (9∶1, v/v) illustrates that, the peptide adopts a stable secondary structure in TFE/H_2_O mixture (Figure S4). The H_α_ CSI prediction is indicative of a helical structure in the N-terminal region comprising residues F2–F18 (Figure S5), although a well-defined three-dimensional structure of the peptide in TFE/H_2_O mixture has not been obtained yet, mainly due to the deficiency of enough unambiguous conformational restraints for the exact structural analysis.

The amphipathic helical conformation is well known to be a crucial factor for many antimicrobial peptides to interact with membranes [45], [46]. On basis of the rough structural analysis described above, it is indicated that the N-terminal region of cantheicidin-BF adopts a typical amphipathic α-helical conformation (Figure S6) as many other cantheicidins.

### Expression profile of tissues

**Figure 4:**
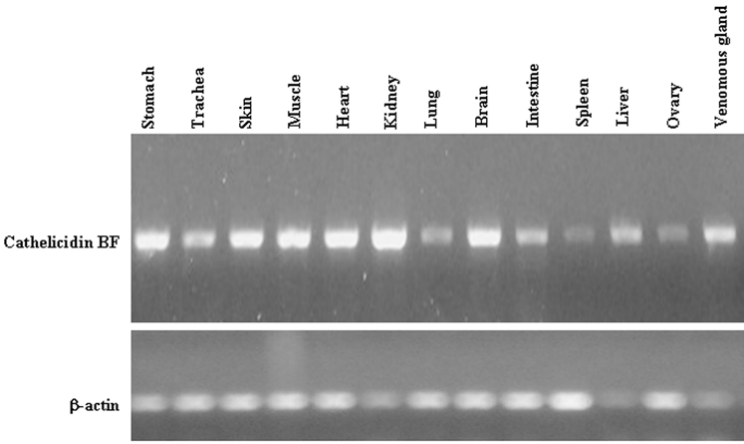
RT-PCR analysis of cathelicidin gene expression pattern in various snake tissues using gene-specific primers with actin as a control.

Using actin as control, expression pattern of cathelicidin-BF was investigated by RT-PCR. Tissue distribution of cathelicidin-BF expression in snake tissues were illustrated in Fig. 4. All the selected tissues including stomach, trachea, skin, muscle, heart, kidney, lung, brain, intestine, spleen, liver, ovary and venomous gland can express this protein.

### Antimicrobial activities

As listed in Table 1, cathelicidin-BF and its analogue, cathelicidin-BF15 showed strong antimicrobial activities against tested microorganisms. Of the 40 tested microorganism strains, cathelicidin-BF exerted potent antimicrobial ability against most of Gram-negative bacteria (either standard strains or clinically isolated drug-resistance strains). For most of *E. coli*, the MICs are lower than 2.3 µg/ml, while ampicillin, benzylpenicillin and ICS are effective only to standard strain with a MIC of 18.7, 37.5 and 0.15 µg/ml respectively. The lowest MIC for *K. pneumoniae* is 0.3 µg/ml, while ampicillin, benzylpenicillin and ICS are effective only to standard strain with a MIC of 150, 18.7 and 9.4 µg/ml respectively. In contrast, most of *S. aureus* are not so sensitive for cathelicidin-BF, only one strain could be killed by cathelicidin-BF with a low MIC (4.7 µg/ml). Another Gram-positive bacteria genus, *Bacillus* also seems to be sensitive for cathelicidin-BF and cathelicidin-BF15. A dangerous clinically isolated strain, *Salmonella typhi* could also be killed by cathelicidin-BF and cathelicidin-BF15 with a low MIC (1.2 µg/ml). Cathelicidin-BF and cathelicidin-BF15 are the same effective to some fungi as bacteria, for example, *C. albicans* ATCC2002 (with a MIC of 4.7 µg/ml).and *P. pastoris* (with a MIC of 0.3 µg/ml). Cathelicidin-BF exerted obvious antimicrobial activity against some saprophytic fungus such as *A. terreus* GIM3.34 (with a MIC of 18.7 µg/ml), *A. niculans* (with a MIC of 4.7 µg/ml), and *C. globosum* (with a MIC of 37.5 µg/ml). All the tested classic antibiotics including Ampicillin, Benzylpenicillin and ICS had no effect on these funguses. Several other cathelicidin-BF analogues, KF1–11 (KFFRKLKKSVK), KF12–19 (KRAKEFFK) and KF20–30 (KPRVIGVSIPF) had no any antimicrobial activity.

**Table 1:** antimicrobial activity comparison of cathelicidin-BF with antibiotics.

Microorganism strains	MIC(ug/ml)					
	BF	BF-15	Amp	Ben	ICS	HDW
*Bacillus subtilis*	9.4	75	0.02	0.004	-	2.3
*Bacillus pumilus*	9.4	-	0.15	0.015	-	-
*Bacillus cereus*	1.2	-	150	150	-	-
*Pseudomonas aeruginosa* ATCC27853	1.2	4.7	150	18.7	9.4	37.5
*P. aeruginosa* (IS, DR)	2.3	37.5	ND	ND	ND	-
*P.aeruginosa* 08031205(IS, DR)	9.4	18.7	ND	ND	ND	-
*P.aeruginosa* 08031014(IS, DR)	18.7	75	ND	ND	ND	-
*Escherichia coli*ATCC25922	2.3	18.7	18.7	37.5	0.15	18.7
*E. coli* 08A852 (IS, DR)	1.2	18.7	ND	ND	ND	-
*E. coli* 08A866(IS, DR)	0.6	18.7	ND	ND	ND	18.7
*E. coli* 08031017 (IS, DR)	2.3	37.5	ND	ND	ND	-
*E. coli* 08032813 (IS, DR)	2.3	>100	ND	ND	ND	-
*E. coli* 08040726 (IS, DR)	0.6	>100	ND	ND	ND	-
*E. coli* 08040722 (IS, DR)	0.6	9.4	ND	ND	ND	-
*Staphylococcus aureus* ATCC2592	4.7	75	0.15	0.03	-	1.2
*S. aureus* ATCC25923	>400	ND	-	-	-	-
*S. aureus* 08A865 (IS, DR)	>400	>200	ND	ND	ND	-
*S. aureus* 08A875 (IS, DR)	75	>200	ND	ND	ND	-
*S. aureus* 08031002 (IS, DR)	>100	>200	ND	ND	ND	-
*S. aureus* 08031013 (IS, DR)	>100	>200	ND	ND	ND	-
*S. aureus* 08032706 (IS, DR)	>100	-	ND	ND	ND	-
*S. aureus* 08032712 (IS, DR)	>100	-	ND	ND	ND	-
*S. aureus* 08032810 (IS, DR)	>100	-	ND	ND	ND	-
*Acinetobacter calcoaceticus*	2.3	-	75	37.5	-	-
*Sphingobacterium siyangense*	9.4	>200	-	-	-	-
*Sacharibacillus kuerlensis*	4.7	4.7	-	-	-	-
*Serratia marcescens* SA	>400	>200	-	-	-	-
*Serratia marcescens* MA	>400	>200	-	-	-	-
*Pseudomonas luteola*	1.2	>200	-	-	-	-
*Salmonella typhi* (IS, DR)	1.2	1.2	-	-	-	-
*Klebsiella pneumoniae* (IS, DR)	4.7	-	-	-	-	-
*K. pneumoniae* 08031012 (IS, DR)	9.4	>200	ND	ND	ND	-
*K. pneumoniae* 08040202 (IS, DR)	0.6	75	ND	ND	ND	-
*K. pneumoniae* 08040724 (IS, DR)	0.3	>100	-	-	-	-
*Enterococcus faecium* (IS, DR)	150	-	-	-	-	-
*Aspergillus terreus* GIM3.34	18.7	ND	ND	ND	ND	-
*Aspergillus niculans*	18.7	ND	ND	ND	ND	-
*Chaetomium globosum*	37.5	ND	ND	ND	ND	-
*Candida albicans* ATCC2002	4.7	18.7	0.3	0.03	-	2.3
*Pichia pastoris*	0.3	9.4	-	-	-	-

MIC: minimal inhibitory concentration. These concentrations represent mean values of three independent experiments performed in duplicates. BF: cathlicidin-BF, BF-15: cathlicidin-BF15, Amp: ampicillin, Ben: benzylpenicillin, ICS: Imipenem and Cilastatin Sodium for Injection, ND: no detectable activity, -: no assay, IS: clinically isolated strain, DR: drug resistance for ampicillin and benzylpenicillin.

The antimicrobial activity of cathelicidin-BF in different solutions was also investigated as listed in Table 2. In 150 mM phosphate buffer solution (PBS) and 150 mM NaCl solution, cathelicidin-BF had stronger antimicrobial activities that in water. It suggested that salts could increase cathelicidin-BF's antimicrobial ability.

**Table 2:** Antimicrobial activity of cathelicidin BF in different solutions.

Microorganism	MIC		
	Water	150 mM NaCl	150 mM PBS
*E. coli*ATCC25922	2.3	2.3	2.3
*P. aeruginosa* ATCC27853	1.2	0.6	0.6
*S. aureus* ATCC2592	4.7	2.4	2.4
*C. albicans* ATCC2002	4.7	2.4	2.4

MIC: minimal inhibitory concentration. These concentrations represent mean values of three independent experiments performed in duplicates.

### Bacteria killing kinetics

Using the antibiotics ICS as a positive control, antibacterial properties of the snake cathelicidin-BF were tested by the colony counting assay. As listed in Table 3 and Table 4, cathelicidin-BF could rapidly exert its antibacterial activities. It just took less than 1 minute to kill all the *E. coli* at the concentration of 1, 5 or 10 times of MIC. The antibacterial activity was proved to be lethal for *E. coli*. *E. coli* was not capable of resuming growth on agar plates after a 6-h treatment with concentrations above the corresponding MICs. In contrast, the antibiotics, ICS could not clean the bacteria within 6 h at the concentration of 1 or 5 times of MIC. Only 10 times MIC of ICS could clean all the *E. coli* within 6 h. Furthermore, *E. coli* treated by 1 time MIC of ICS was capable of resuming growth during 6 h.

**Table 3:** Bacterial killing kinetics of cathelicidin-BF against *E. coli*.

Amount of bacteria co-cultured with different samples for different time (CFU)					
Time (h)	0	0.1	1	3	6
Samples					
BFx1	50	0	0	0	0
BFx5	50	0	0	0	0
BFx10	50	0	0	0	0
ICSx1	50	36	12	224	1082
ICSx5	50	35	0.3	0.7	7
ICSx5	50	19	0.3	0.7	0
0.9% salt water	50	45	234	2341	14109

BF: cathlicidin-BF, CFU: colony forming unit, ICS: Imipenem and Cilastatin Sodium for Injection, ×1, ×5 and ×10: 1, 5 and 10 times

**Table 4:** Bacterial killing kinetics of cathelicidin-BF and HDW against *E. coli*.

Amount of bacteria co-cultured with different samples for different time (CFU)					
Time (min)	0	0.1	1	10	30
Samples					
BFx1	50	32	0	0	0
BFx5	50	20	0	0	0
BFx10	50	10	0	0	0
HDWx1	50	49	14.7	0	0
HDWx10	50	38	3.7	0	0
0.9% salt water	50	69	56	66.7	68.7

BF: cathlicidin-BF, CFU: colony forming unit, ×1, ×5 and ×10: 1, 5 and 10 times, HDW: A amphibian antimicrobial peptide from *Rana nigrovittata*.

In order to compare properties with other antimicrobial peptide, the frog antimicrobial peptide HDW was used as a control. Their bacteria killing kinetics during 30 min was listed in Table 4. Although HDW had a rapid bacteria killing ability, cathelicidin-BF is faster to clean *E. coli* than HDW. Cathelicidin-BF just took less than 1 minute to clean *E. coli*, while HDW took several minutes.

### Hemolysis, cytotoxicity, serum stability

Cathelicidin-BF had little hemolytic activity on human red blood cells even with peptide concentrations up to 400 µg/ml. At the same concentration, cathelicidin-BF was neither cytotoxic for mouse macrophage (RAW264.7) nor for human liver tumor cell (HepG_2_) (data not shown). Thus, it showed considerable selectivity for microorganisms over mammalian cells *in vitro*.

Serum stability was checked by incubating 100 µg/ml cathelicidin-BF and cathelicidin-BF15 with 90% fresh normal human serum at 37°C for 0, 1, 2, 3, 6, 10 and 24 hours. For cathelicidin-BF, antimicrobial activities against *E. coli* 08A866 could not be detected after 3 h-incubation, while cathelicidin-BF15 could keep its antimicrobial activity up to 10 h in 90% fresh normal human serum. Cathelicidin-BF15 seems to be more stable than cathelicidin-BF in serum.

## Discussion

Antimicrobial peptides (AMPs) and their precursor molecules form a central part of biological immunity. For the species which lack adaptive immunity, AMPs play a key role to defense microorganism infection. For their capacity to rapidly inactive infectious agents and to probably inhibit the emergence of drug resistance, AMPs have attracted considerable attention, especially for the treatment of antibiotic-resistant pathogens. The most two important AMP families, defensin and cathelicidin have been found in mammalians, birds and fish. Coincidently, both defensin and cathelicidin have not been found in both reptiles and amphibians although a beta-defensin-like protein with unusual disulfide connectivity (C1–C6/C2–C5/C3–C4), which is different from other the vertebrate beta-defensins, has been identified from a marine turtle [47], [48]. Several hundreds of gene-encoded AMPs have been found from amphibians [12], [39]–[42], [49]. Only a few peptides or proteins from reptiles have been found to exert antimicrobial activities, and most of them are phospholipases A_2_ or its derivatives [34], [50]–[52], and L-amino acid oxidase [53].

In the attempt to find AMPs from the snake venoms of *B. fasciatus*, which is a rich source of biological peptides or proteins with therapeutic potential, an AMP, cathelicidin-BF has been isolated and characterized. By screening the cDNA, cathelicidin-BF was unexpectedly found to be a C-terminus of a cathelicidin. The cathelicidin-BF precursor is composed of 191 amino acid residues with conserved cathelin domain that was flanked by signal peptide and by mature cathelicidin-BF. A conserved cleavage site (Valine^157^) for elastase in the processing and maturation of bovine, porcine and chicken cathelicidins [30] is also existed in the cathelicidin-BF precursor, suggesting that the snake cathelicidin is possibly processed by elastase-like proteases. Based on the hypothesis, cathelicidin-BF precursor should release a 34-aa peptide fragment (KRFKKFFRKLKKSVKKRAKEFFKKPRVIGVSIPF), which has a 4-aa (KRFK) extension at the N-terminus of the 30-aa cathelicidin-BF (KFFRKLKKSVKKRAKEFFKKPRVIGVSIPF). Two reasons may explain the length difference between the predicted 34-aa C-terminal peptide and the purified 30-aa cathelicidin-BF in this case: 1, the predicted cleavage site is right, and the purified 30-aa cathelicidin-BF is from the further processing of the 34-aa peptide; 2, Valine^157^ in the cathelicidin-BF precursor is not the exact protease cleavage site. In fact, some cathelicidins is not cleaved by elastase to release C-terminal active peptide fragments as mentioned in this “introduction”. An obvious feather of cathelicidin-BF is that there is a high density (40%) of basic amino acid residues in its sequence. As some other cathelicidins [30], there are multiple aromatic amino acid residues in cathelicidin-BF's sequence (5 Phenylalanines). An atypical feature of cathelicidin-BF precursor is that an acidic domain insertion (EEGEQKQEEGNEEEKEVEEEEQEED EKD) is located between the cathelin domain and the mature cathelicidin-BF. This region potentially could affect preproprotein net charge, stability, activity or processing. The similar acidic regions are also found in amphibian antimicrobial precursors, which are located between the signal peptide domains and the mature antimicrobial domains [39]–[42]. Amphibian acidic regions act as a role to neutralize the positive charge of the mature antimicrobial domains and to avoid possible toxicity of the precursor proteins.

The data of antimicrobial testing indicated that cathelicidin-BF is clearly among the most potent cathlicidins discovered to date. Among the 40 strains of tested microorganisms, 15 strains could be killed by cathelicidin-BF at <0.6 µM. For a variety of microorganisms, cathelicidin-BF had better antimicrobial ability than ampicillin, benzylpenicillin and ICS. Cathelicidin-BF's obvious ability to kill some saprophytic fungus such as *A. terreus*, *A. niculans* and *C. globosum* is also very interesting. It may be used as agricultural antibiotics against plant or food pathogenic microorganisms. To our knowledge, this is the first report of cathelicidin's antimicrobial activities against saprophytic fungus. In addition, cathelicidin-BF had very rapid microbe-killing efficacy. Cathelicidin-BF could kill *E. coli* within one minute at the dose of one time MIC. All the results suggest that cathelicidin-BF is an excellent candidate for clinical or agricultural antibiotics.

## Supporting Information

Materials and Methods S1 Detail materials and methods
(0.03 MB DOC)

Figure S1 Purification of the cathelicidin from snake venom. (a) Gel filtration chromatography. Sephadex G-50 column (2.6 cm×100 cm), equilibrated and developed with 50 mM Tris-HCl plus 50 mM NaCl (pH 7.8) at a flow rate of 0.3 ml/min, fractions were collected. (b) Cation-exchange chromatography. CM-Sephadex C-25 column (16 cm×40 cm) elution was achieved with a liner NaCl gradient, at a flow rate of 1 ml/min. (c) RP-HPLC chromatography. C4 reverse phase column, equilibrated with 0.1% (v/v) TFA/water, elution was performed with an acetonitrile liner gradient at a flow rate of 0.7 ml/min. The purified peptide with antimicrobial activity is indicated by an arrow.
(0.17 MB TIF)

Figure S2 Electrospray ionization mass spectrometry analysis of the RP-HPLC peak containing antimicrobial activity.
(0.11 MB TIF)

Figure S3 Circular dichroism spectra recorded on cathelicidin-BF in different solvent environments. (A) a∼e : in SDS micelles of 0, 30, 60, 90, 120 mM; (B) a∼e: in TFE/H_2_O mixtures of 1∶9, 3∶7, 5∶5, 7∶3, 9∶1 (v/v).
(0.13 MB TIF)

Figure S4 HN-HN regions of 2D ^1^H-^1^H NOESY spectra recorded on cathelicidin-BF in H_2_O (left) and in TFE/H_2_O mixture (9∶1, v/v) (right).
(0.12 MB TIF)

Figure S5 Hα CSI prediction for the cathelicidin-BF peptide in TFE/H_2_O mixture (9∶1, v/v). h: helix; c: coil.
(0.16 MB TIF)

Figure S6 The opposing position of the hydrophilic and hydrophobic side chains can be seen in this end-on representation of the α-helix in the N-terminal region of cathelicidin-BF.
(0.18 MB TIF)

Table S1 Contents of helical structures of cathelicidin in TFE/H_2_O mixtures or in SDS micelles measured by CD.
(0.03 MB DOC)

Table S2 ^1^H chemical shifts of cathelicidin-BF in TFE/H_2_O mixture (9∶1, v/v) at 298 K
(0.07 MB DOC)
