# Supplementary material for: Snake Cathelicidin from Bungarus fasciatus Is a Potent Peptide Antibiotics
Source: PLoS One. 2008 Sep 16;3(9):e3217. doi: 10.1371/journal.pone.0003217 (PMC2528936; doi:10.1371/journal.pone.0003217)
Supplement: Materials and Methods S1 — Detail materials and methods (0.03 MB DOC) [file pone.0003217.s001.doc]

**Materials and Methods S1**

**Antimicrobial assays**

Standard and clinically isolated microorganism strains were used for antimicrobial assays. Bacteria were first grown in LB (Luria-Bertani) broth to an OD_600 nm_ of 0.8. A 10 μl aliquot of the bacteria was then taken and added to 8 ml of fresh LB broth with 0.7% agar and poured over a 90 mm Petri dish containing 25 ml of 1.5% agar in LB broth. After the top agar hardened, a 20 μl aliquot of the test sample filtered on a 0.22 μm Millipore filter was dropped onto the surface of the top agar and completely dried before being incubated overnight at 37^o^C. If the sample examined had antimicrobial activity, a clear zone would be formed on the surface of the top agar representing inhibition of bacterial growth. Minimal inhibitory concentration (MIC) was determined in liquid LB medium by incubating the bacteria in LB broth with variable amounts of the sample tested. The MIC at which no visible growth occurred was recorded. In the assays of anti-fungi activity, for strains of *Aspergillus terreus,* *A. niculans*, and *Chaetomium globosum*, the fungal spore concentration was first counted under a microscope. The fungi with an initial concentration of 10^5^ spores/ml were cultured with variable amounts of the sample tested in yeast extract-peptone-dextrose broth.

**Bacteria killing kinetics**

The bacterial effect of cathelicidin-BF against *E. coli* 08A866 was tested at 1, 5 and 10 times MIC, Imipenem and Cilastatin Sodium for Injection (ICS) at 1, 5 and 10 times MIC and frog antimicrobial peptide HDW at 1 and 10 times MIC, respectively. Fresh overnight colonies were added to a total of 20 ml LB, and incubated on a 35 ^o^C water bath. After one hour of incubation, 1 ml broth from each flask was replaced with the test solution. The colony counting was performed at different time after addition of the test solutions as described by by Mygind *et al* [39].

**Hemolysis, cytotoxicity, serum stability**

Hemolysis assays were undertaken using human red blood cells in liquid medium as our previous reoprts [35-38]. Serial dilutions of the peptide were used, and after incubation at 37^o^C for 30 min, the cells were centrifuged and the absorbance in the supernatant was measured at 540 nm. Maximum hemolysis was determined by adding 1% Triton X-100 to a sample of cells. Cytotoxicity was measured according the methods described by Mygind *et al*, using mouse macrophage (RAW264.7) and tumor cell (HepG_2_). Serum stability was checked by incubating 100 μg/ml cathelicidin-BF with 90% fresh normal human serum at 37 ^o^C for 0, 1, 2, 3, 6, and 24 hours. Residual antimicrobial activities were measured by performing radial diffusion assays with *E. coli* 08A866 as described by Mygind *et al* [39].
