# Supplementary figures and images for: Snake Cathelicidin from Bungarus fasciatus Is a Potent Peptide Antibiotics
Source: PLoS One. 2008 Sep 16;3(9):e3217. doi: 10.1371/journal.pone.0003217 (PMC2528936; doi:10.1371/journal.pone.0003217)

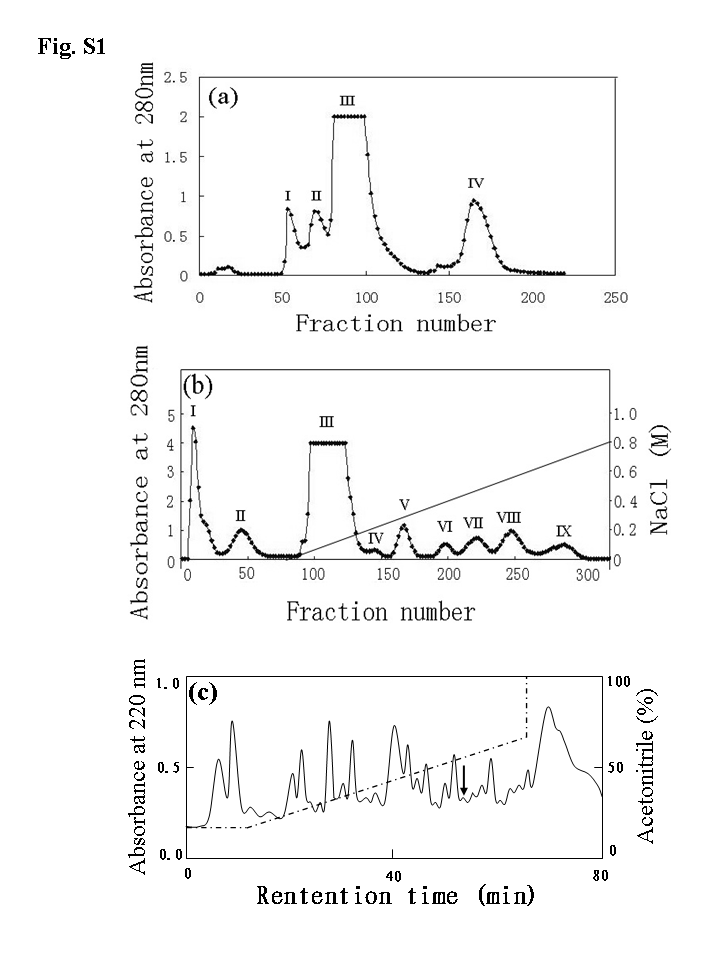

Supplement: Figure S1 — Purification of the cathelicidin from snake venom. (a) Gel filtration chromatography. Sephadex G-50 column (2.6 cm×100 cm), equilibrated and developed with 50 mM Tris-HCl plus 50 mM NaCl (pH 7.8) at a flow rate of 0.3 ml/min, fractions were collected. (b) Cation-exchange chromatography. CM-Sephadex C-25 column (16 cm×40 cm) elution was achieved with a liner NaCl gradient, at a flow rate of 1 ml/min. (c) RP-HPLC chromatography. C4 reverse phase column, equilibrated with 0.1% (v/v) TFA/water, elution was performed with an acetonitrile liner gradient at a flow rate of 0.7 ml/min. The purified peptide with antimicrobial activity is indicated by an arrow. (0.17 MB TIF) [file pone.0003217.s002.tif]

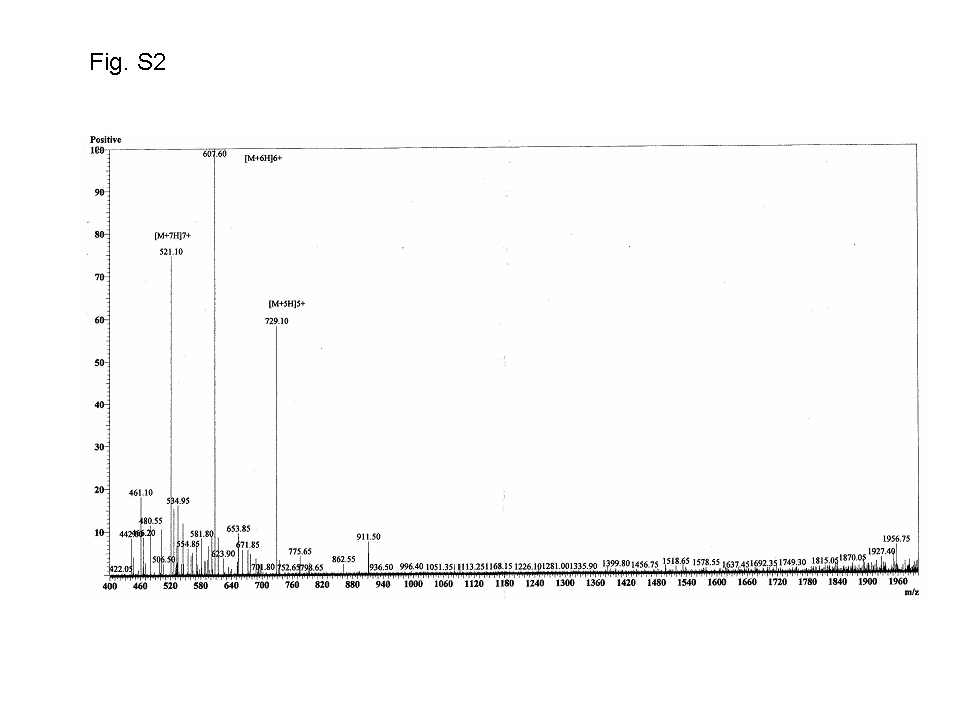

Supplement: Figure S2 — Electrospray ionization mass spectrometry analysis of the RP-HPLC peak containing antimicrobial activity. (0.11 MB TIF) [file pone.0003217.s003.tif]

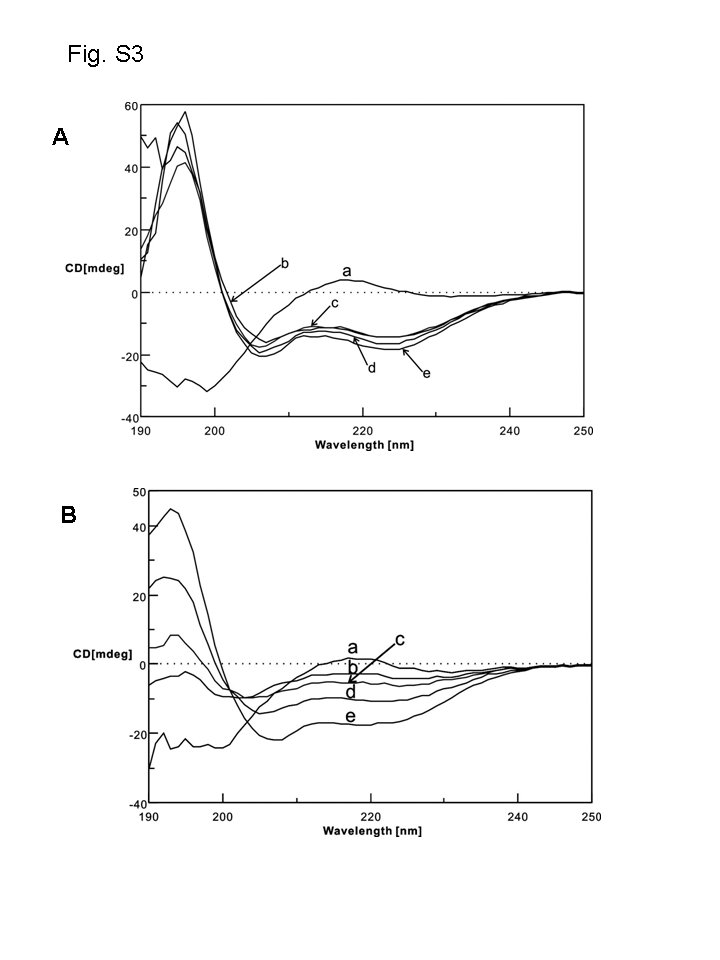

Supplement: Figure S3 — Circular dichroism spectra recorded on cathelicidin-BF in different solvent environments. (A) a∼e : in SDS micelles of 0, 30, 60, 90, 120 mM; (B) a∼e: in TFE/H2O mixtures of 1∶9, 3∶7, 5∶5, 7∶3, 9∶1 (v/v). (0.13 MB TIF) [file pone.0003217.s004.tif]

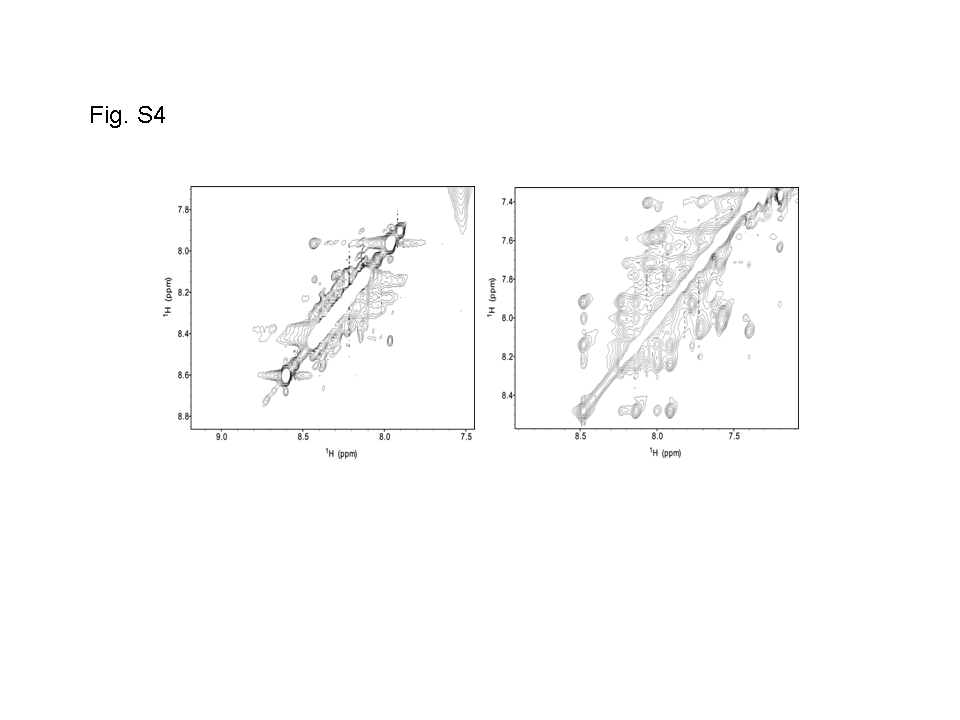

Supplement: Figure S4 — HN-HN regions of 2D 1H-1H NOESY spectra recorded on cathelicidin-BF in H2O (left) and in TFE/H2O mixture (9∶1, v/v) (right). (0.12 MB TIF) [file pone.0003217.s005.tif]

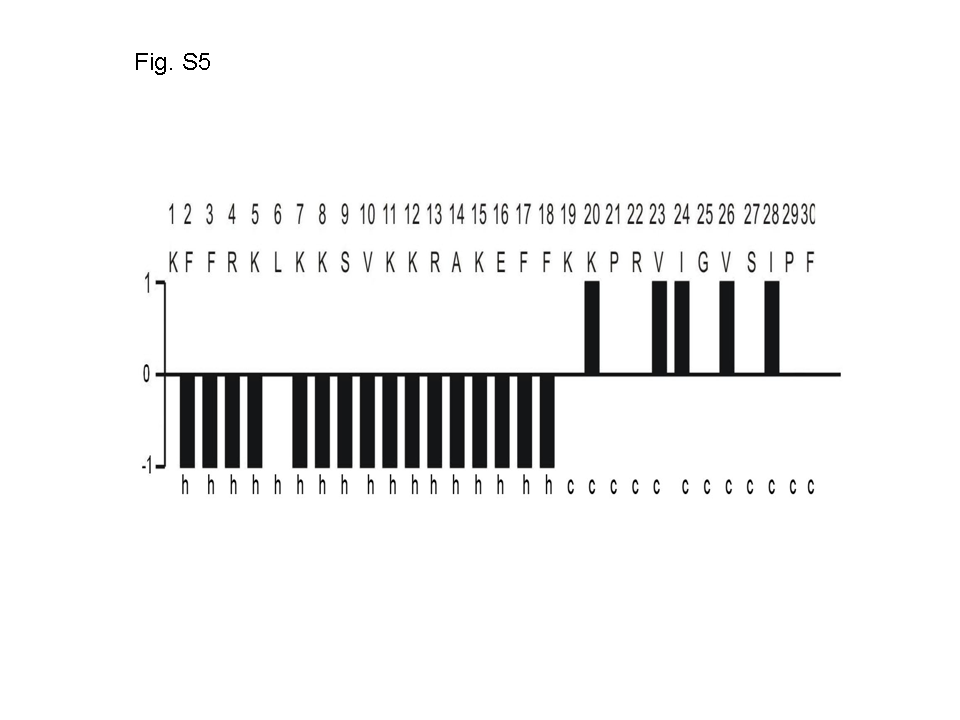

Supplement: Figure S5 — Hα CSI prediction for the cathelicidin-BF peptide in TFE/H2O mixture (9∶1, v/v). h: helix; c: coil. (0.16 MB TIF) [file pone.0003217.s006.tif]

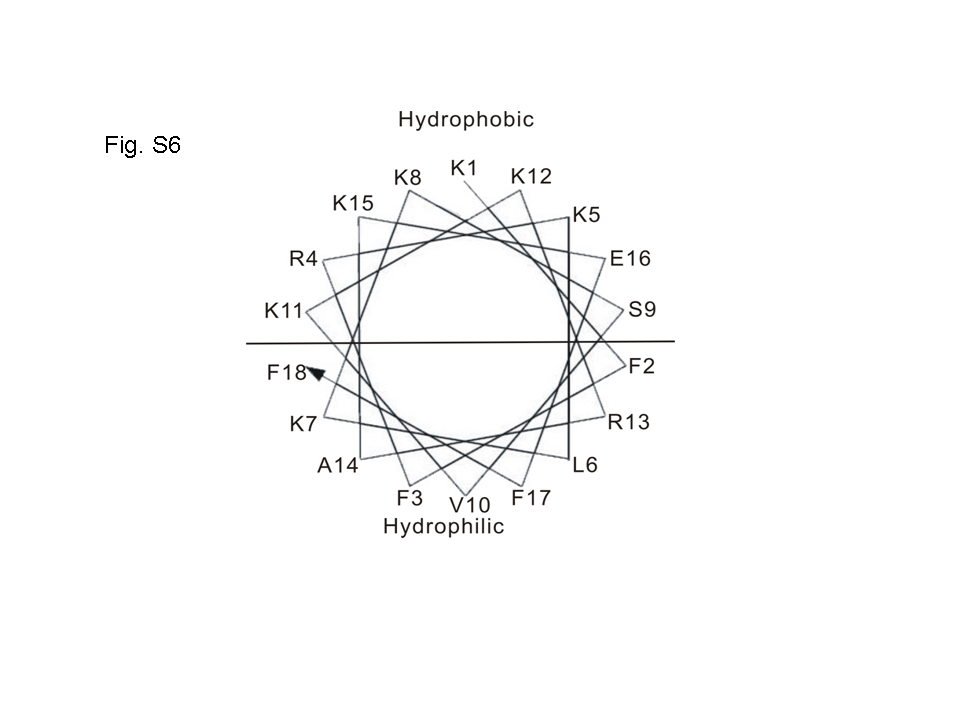

Supplement: Figure S6 — The opposing position of the hydrophilic and hydrophobic side chains can be seen in this end-on representation of the α-helix in the N-terminal region of cathelicidin-BF. (0.18 MB TIF) [file pone.0003217.s007.tif]
