# Supplementary material for: Snake Cathelicidin from Bungarus fasciatus Is a Potent Peptide Antibiotics
Source: PLoS One. 2008 Sep 16;3(9):e3217. doi: 10.1371/journal.pone.0003217 (PMC2528936; doi:10.1371/journal.pone.0003217)
Supplement: Table S1 — Contents of helical structures of cathelicidin in TFE/H2O mixtures or in SDS micelles measured by CD. (0.03 MB DOC) [file pone.0003217.s008.doc]

Table S1 Contents of helical structures of cathelicidin in TFE/H_2_O mixtures or in SDS micelles measured by CD.

| TFE/H_2_O (v/v) | Helix (%) | SDS (mM) | Helix (%) |
| --- | --- | --- | --- |
| 1:9 | 0 | 0 | 0 |
| 3:7 | 5 | 30 | 38 |
| 5:5 | 13 | 60 | 31 |
| 7:3 | 39 | 90 | 27 |
| 9:1 | 42 | 120 | 77 |
