# Supplementary material for: Snake Cathelicidin from Bungarus fasciatus Is a Potent Peptide Antibiotics
Source: PLoS One. 2008 Sep 16;3(9):e3217. doi: 10.1371/journal.pone.0003217 (PMC2528936; doi:10.1371/journal.pone.0003217)
Supplement: Table S2 — 1H chemical shifts of cathelicidin-BF in TFE/H2O mixture (9∶1, v/v) at 298 K (0.07 MB DOC) [file pone.0003217.s009.doc]

Table S2 ^1^H chemical shifts of cathelicidin-BF in TFE/H_2_O mixture (9:1, v/v) at 298 K

| Residue | HN | H_α_ | H_β_ | H_γ_ | others |
| --- | --- | --- | --- | --- | --- |
| Lys1 |  | 4.380 | 1.911 | 1.468 | 1.649, 3.039 |
| Phe2 | 7.287 | 4.646 | 3.229, 3.096 |  | 7.044, 7.277 |
| Phe3 | 7.639 | 4.339 | 3.185, 3.074 |  | 7.206, 7.374 |
| Arg4 | 7.931 | 4.029 | 1.910 | 1.664 | 3.238, 1.763 |
| Lys5 | 7.777 | 4.018 | 1.914 | 1.442,1.548 | 1.716, 2.982 |
| Leu6 |  |  |  |  | 0.947 |
| Lys7 | 7.996 | 4.028 | 1.997, 1.933 | 1.478 | 1.720 |
| Lsy8 | 8.178 | 4.002 | 1.992 | 1.488 | 1.678 |
| Ser9 | 8.074 | 4.237 | 4.035 |  |  |
| Val10 | 8.137 | 3.716 | 2.239 | 1.096,0.922 |  |
| Lys11 | 8.234 | 4.029 | 2.001 | 1.488 | 1.567 |
| Lys12 | 8.141 | 3.950 | 1.893 | 1.424 | 1.718, 1.653 |
| Arg13 | 7.919 | 4.117 | 1.998 | 1.648 | 3.197, 1.827 |
| Ala14 | 8.480 | 1.596 |  |  |  |
| Lys15 | 8.136 | 4.145 | 2.074, 2.008 | 1.553 | 1.756, 3.021 |
| Glu16 | 8.004 | 4.128 | 2.180, 2.108 | 2.473,2.400 |  |
| Phe17 | 8.200 | 4.262 | 3.132, 3.014 |  | 6.825, 7.180 |
| Phe18 | 8.067 | 4.446 | 3.327, 3.114 |  | 7.043 |
| Lys19 | 7.718 | 4.266 | 1.925 | 1.558, 1.475 | 1.725, 3.129, 2.999 |
| Lys20 | 7.595 | 4.557 | 2.352, 2.154 |  |  |
| Pro21 |  | 4.371 | 2.260, 2.077 | 1.993, 1.882 | 3.818, 3.604 |
| Arg22 | 7.829 | 4.364 | 1.856, 1.792 | 1.654 | 3.139 |
| Val23 | 7.708 | 4.133 | 2.084 | 0.939 |  |
| Ile24 | 7.715 | 4.105 | 1.864 | 1.207 | 0.944 |
| Gly25 | 8.029 | 4.104 | 3.804 |  |  |
| Val26 | 7.579 | 4.204 | 2.094 | 0.951 |  |
| Ser27 | 7.974 | 4.523 | 3.881, 3.847 |  |  |
| Ile28 | 7.567 | 4.528 | 1.834 | 1.537,1.155 | 0.921 |
| Pro29 |  | 4.439 | 2.057, 1.954 | 1.912,1.806 | 3.743, 3.536 |
| Phe30 | 7.007 | 4.456 | 3.171, 2.992 |  | 7.217, 7.288 |
